# Potent Protein Glycation Inhibition of Plantagoside in *Plantago major* Seeds

**DOI:** 10.1155/2014/208539

**Published:** 2014-05-07

**Authors:** Nobuyasu Matsuura, Tadashi Aradate, Chihiro Kurosaka, Makoto Ubukata, Shiho Kittaka, Yuri Nakaminami, Kanae Gamo, Hiroyuki Kojima, Mitsuharu Ohara

**Affiliations:** ^1^Department of Life Science, Faculty of Science, Okayama University of Science, 1-1 Ridai-cho, Kita-ku, Okayama 700-0005, Japan; ^2^University of Toyama, 2630 Sugitani, Toyama 930-0194, Japan; ^3^Biotechnology Research Center, Toyama Prefectural University, 5180 Kurokawa, Kosugi-machi, Imizu-gun, Toyama 939-0398, Japan; ^4^Graduate School of Agriculture, Hokkaido University, Kita-9, Nishi-9, Kita-ku, Sapporo 060-8589, Japan; ^5^Department of Pharmaceutical Science, Graduate School of Medicine, Density and Pharmaceutical Sciences, Okayama University, 1-1-1 Tsushima-naka, Kita-ku, Okayama 700-8530, Japan; ^6^Ichimaru Pharcos Co., Ltd., 318-1 Asagi, Motosu-shi, Gifu 501-0475, Japan

## Abstract

Plantagoside (5,7,4′,5′-tetrahydroxyflavanone-3′-*O*-glucoside) and its aglycone (5,7,3′,4′,5′-pentahydroxyflavanone), isolated from a 50% ethanol extract of *Plantago major* seeds (Plantaginaceae), were established to be potent inhibitors of the Maillard reaction. These compounds also inhibited the formation of advanced glycation end products in proteins in physiological conditions and inhibited protein cross-linking glycation. These results indicate that *P. major* seeds have potential therapeutic applications in the prevention of diabetic complications.

## 1. Introduction

Nonenzymatic protein glycation in the body leads to vascular and renal complications of diabetes [1]. Diabetic patients tend to accumulate glycated proteins in their body tissues because their blood glucose concentration is higher than that in healthy individuals. The initial chemical modification step is the reaction between the free amino group of proteins and carbonyl group of glucose, which leads to the formation of fructosamines via Schiff bases, followed by the Amadori rearrangement. The fructosamines are successively oxidized, dehydrated, and condensed to form cross-linked proteins and eventually advanced glycation end products (AGEs). Some AGEs have been isolated, and their structures have been elucidated (e.g., pyrraline [2], *N* ^e*ε*^-(carboxymethyl)lysine [3], pentosidine [4], 2-(2-furoyl)-4(5)-(2-furanyl)-1H-imidazole [5], and crossline [6]).

Various attempts have been made to identify effective glycation inhibitors. Aminoguanidine has the capacity to prevent the diabetes-induced formation of AGEs, including the inhibition of protein cross-linking [7]. Aspirin [8] as well as vitamin B_6_ [9], taurine [10], quercetin [11], and other natural inhibitors have also been reported. To identify effective glycation inhibitors, we devised an improved screening system, which was reported in a previous study [12]. In the present report, we describe the screening results and the differences in the inhibitory mechanisms of plantagoside and aminoguanidine.

## 2. Materials and Methods

### 2.1. Materials

*Plantago major* seeds were purchased from Ichimaru Pharcos Co., Ltd. (Gifu Japan). Plantagoside (5,7,4′,5′-tetrahydroxyflavanone-3′-*O*-glucoside) (**1**) and its aglycone (5,7,3′,4′,5′-pentahydroxyflavanone) (**2**) were isolated from a 50% ethanol extract of* P. major* seeds (Plantaginaceae). 5,7-Dihydroxy-3′,4′,5′-trimethoxyflavone (**3**), myricetin (5,7,3′,4′,5′-pentahydroxyflavonol) (**6**), and dihydromyricetin (5,7,3′,4′,5′-pentahydroxyflavanonol) (**7**) were purchased from Funakoshi Co., Ltd. (Japan). Aminoguanidine hydrochloride, N-*α*-acetyl-lysine, and N-*α*-acetyl-arginine were purchased from Sigma-Aldrich (USA). Cell-matrix type I-C collagen was purchased from Asahi Techno Glass (Japan). All the other chemicals were purchased from Nakarai Tesque, Inc. (Japan).

### 2.2. Evaluation of Glycation

#### 2.2.1. Fluorometric Analysis

Our improved Maillard reaction inhibitor screening system was used for bioassay-guided fractionation and for isolating active compounds in the present study [12]. In brief, a reaction mixture (500 *μ*L) was prepared in a plastic tube (1.5 mL) that comprised bovine serum albumin (BSA) (400 *μ*g) and glucose (200 mM), with/without an inhibitor or plant extract (10 *μ*L), in 50 mM phosphate buffer, pH 7.4. The reaction mixture was heated at 60°C on a heating block for 30 h. The blank sample and the unreacted solution without an inhibitor or plant extract were maintained at 4°C until measurement. After cooling, an aliquot (100 *μ*L) was transferred to a new plastic tube (1.5 mL), and 100% w/v trichloroacetic acid (TCA) (10 *μ*L) was added to each tube. The supernatant containing glucose, inhibitor, and interfering substances was discarded after agitation and centrifugation (15,000 rpm, 4°C, 4 min). The precipitate containing AGEs-BSA was then dissolved in alkaline PBS (400 *μ*L). Alkaline PBS contained NaCl (137 mM), Na_2_HPO_4_ (8.1 mM), KCl (2.68 mM), and KH_2_PO_4_ (1.47 mM) and was adjusted to pH 10 with 0.25 N NaOH. The fluorescence intensity (ex. 360 nm, em. 460 nm) related to AGEs-protein was measured using a Cytofluor II fluorescence multiwell plate reader (PersSeptive Biosystems, USA). The true inhibition activity was estimated by subtracting the quenching effect from the apparent inhibitory activity. The apparent inhibitory activity was calculated using the method described above. The quenching effect was measured using the same sample dissolved in alkaline PBS after TCA treatment of the mixed plant extract solution (2 and 100 *μ*L) after incubating the control solution for 30 h without the inhibitor or plant extract. The Maillard reaction was performed for 96 h without TCA precipitation using N-*α*-acetyl-lysine or N-*α*-acetyl-arginine instead of BSA as the substrate.

#### 2.2.2. Protein Cross-Linking

The reaction mixtures (1 mL each), containing lysozyme (5.0 mg) and ribose (20 mM), were prepared in phosphate buffer (100 mM, pH 7.4). Each reaction mixture was sterile filtered into a plastic tube and placed in an incubator at 37°C for 1 week. Sodium dodecyl sulfate polyacrylamide gel electrophoresis (SDS-PAGE) was performed according to the method described by Laemmli [13]. Aliquots (10 *μ*L each) of the reaction mixtures were heated at 95°C with a buffer (2 *μ*L) containing 4% SDS, 10% *β*-mercaptomethanol, 20% glycerol, 0.2 M glycine, and 1% methylene blue in 0.5 M Tris buffer (pH 6.7). After electrophoresis, the gel was stained with Coomassie Brilliant Blue (CBB), destained, and dried.

### 2.3. Purification and Structural Determination of Plantagoside and 5,7,3′,4′,5′-Pentahydroxyflavanone

*P. major* seeds (213 g) were extracted with 50% ethanol (2.2 L) for 1 week at room temperature. A crude extract (7.6 g) was obtained by concentration* in vacuo* and was suspended in H_2_O (1 L) before the solution was extracted with chloroform (1 L). The aqueous layer was further extracted with ethylacetate (2 L). The resulting extracts were concentrated to produce a crude oil (0.64 g), which was subjected to reversed-phase high-performance liquid chromatography (HPLC). The HPLC conditions were as follows: flow rate: 4.6 mL/min; detector: UV 210 nm; solvent: methanol: H_2_O (30 : 70); column oven temperature: 40°C; and column: Mightysil RP-18, 10 × 250 mm. Two active fractions (fractions 1 and 2) were obtained. Further purification of the active fractions using HPLC yielded plantagoside (**1**) (148 mg) and 5,7,3′,4′,5′-pantahydroxyflavanone (**2**) (23 mg).

Plantagoside (**1**): negative-ion FAB-MS* m/z*: 465 [M-H]^+^; ^1^H-NMR (400 MHz, DMSO-*d*_6_ + D_2_O) *δ*: 2.70 (1H, dd, *J* = 17 and 3 Hz, H-3), 3.07 (1H, dd, *J* = 17 and 12 Hz, H-3), 4.64 (1H, d, *J* = 8 Hz, H-1 of glucose), 5.32 (1H, dd, *J* = 12 and 3 Hz, H-2), 5.81 (1H, d, *J* = 2 Hz, H-6), 5.82 (1H, d, *J* = 2 Hz, H-8), 6.56 (1H, d, *J* = 2 Hz, H-6′), 6.70 (1H, d, *J* = 2 Hz, H-2′); ^13^C-NMR (100 MHz, DMSO-*d*_6_ + D_2_O) *δ*: 41.9 (C-3), 60.7 (glc-6), 69.8 (glc-4), 73.3 (glc-2), 75.9 (glc-5), 77.1 (glc-3), 78.4 (C-2), 95.0 (C-8), 95.8 (C-6), 101.7 (C-10), 102.4 (glc-1), 106.4 (C-6′), 109.2 (C-2′), 128.6 (C-1′), 135.2 (C-4′), 145.7 (C-3′), 145.8 (C-5′), 162.8 (C-9), 163.4 (C-5), 166.7 (C-7), 196.1 (C-4).

5,7,3′,4′,5′-Pentahydroxyflavanone (**2**): negative-ion FAB-MS* m/z*: 303 [M-H]^+^; ^1^H-NMR (400 MHz, DMSO-*d*_6_) *δ*: 2.70 (1H, dd, *J* = 17 and 3 Hz, H-3), 3.07 (1H, dd, *J* = 17 and 12 Hz, H-3), 5.32 (1H, dd, *J* = 12 and 3 Hz, H-2), 5.81 (1H, d, *J* = 2 Hz, H-6), 5.82 (1H, d, *J* = 2 Hz, H-8), 6.56 (2H, s, H-2′ and 6′).

### 2.4. Synthesis of 5,7,3′,4′,5′-Pentahydroxyflavone (****4****)

5,7-Dihydroxy-3′,4′,5′-trimethoxyflavone (**3**; 15 mg) was added to AlCl_3_ (8 g) and NaCl (1.4 g) and melted by heating at 180°C. After 10 min, the reaction mixture was cooled and dissolved in 2 N HCl, and** 3** (2 mg) was extracted with ethylacetate. ^1^H-NMR (400 MHz, acetone-*d*_6_) *δ*: 6.20 (1H, s, H-3), 6.46 (2H, brs, H-6, 8), 7.10 (2H, s, H-2′, 6′), 12.98 (1H, brs, C_5_-OH).

## 3. Results and Discussion

**Figure 1:**
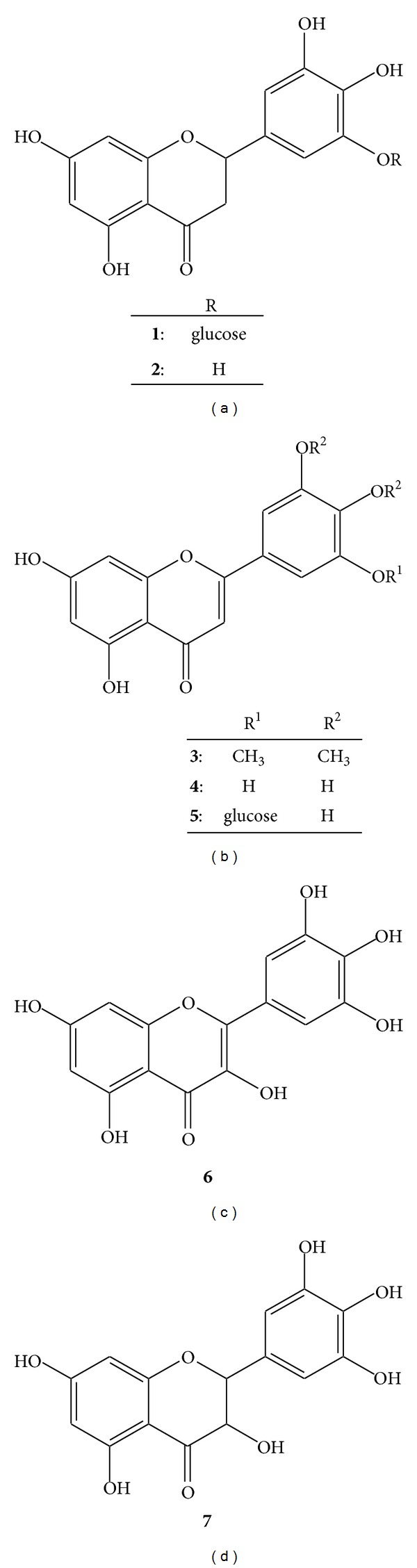
The structures of the flavonoids that inhibited glycation.** 1**: Plantagoside (R = glucose),** 2**: 5,7,3′,4′,5′-pentahydroxyflavanone (R = H),** 3**: 5,7-dihydroxy-3′,4′,5′-trimethoxyflavone (R^1^ = R^2^ = CH_3_),** 4:** 5,7,3′,4′,5′-pentahydroxyflavone (R^1^ = R^2^ = H),** 5:** 5,7,4′,5′-tetrahydroxyflavone-3′-*O*-glucoside (R^1^ = glucose, R^2^ = H),** 6**: myricetin, and** 7:** dihydromyricetin.

We examined 200 natural plant extracts to determine their inhibitory effects on the Maillard reaction and obtained satisfactory results with an extract of* P. major* seeds. The active compounds were characterized as plantagoside (**1**) and its aglycone (**2**) after analyzing the FAB-MS, ^1^H-NMR, and ^13^C-NMR spectral data (Figure 1). Flavanones** 1** and** 2** were first isolated from* P. asiatica* var.* japonica *[14] and* Helichrysum bracteatum *[15]. *α*-Mannosidase inhibitory activity has also been reported for** 1 **[16]. The Maillard reaction inhibitory activities of** 1** (IC_50_, 1.2 *μ*M) and** 2** (IC_50_, 18.0 *μ*M) were approximately 83- and 5.5-times stronger, respectively, than that of aminoguanidine (IC_50_, 100 *μ*M), which was used as a known Maillard reaction inhibitor [17] in our established assay system [12]. It has been reported that some natural flavonoids, that is, baicalin, baicalein [17], quercetin [11], and maritimein [18], inhibit the Maillard reaction, and these compounds also showed inhibitory activities when our assay system was used (baicalin (IC_50_, 25.0 *μ*M), baicalein (IC_50_ > 100 *μ*M), quercetin (IC_50_, 1.4 *μ*M), and maritimein (IC_50_, 10.0 *μ*M)). These results indicated that** 1** had the highest inhibitory activity among the known isolated natural products.

To clarify the inhibitory activities of** 1 **and** 2** in physiological conditions, collagen, one of the major proteins in the human body, was used as a substrate instead of BSA. The incubation temperature was changed to 37°C from 60°C. The inhibitory activities (IC_50_) were 14 *μ*M for** 1**, 42 *μ*M for** 2**, and 2500 *μ*M for aminoguanidine. The inhibitory activity of each compound was 2–25-times lower than that obtained using the original conditions. When BSA was used as the substrate and the reaction temperature was high, the rank order of the inhibitory activity of each compound remained unchanged. These results demonstrated that our improved Maillard reaction inhibitor screening method is very useful for bioassay-guided isolation of effective compounds and evaluation of the inhibitory activities of such compounds. Plantagoside was identified using this assay, and it was found to inhibit the formation of AGEs in physiological conditions at a lower concentration.

**Figure 2:**
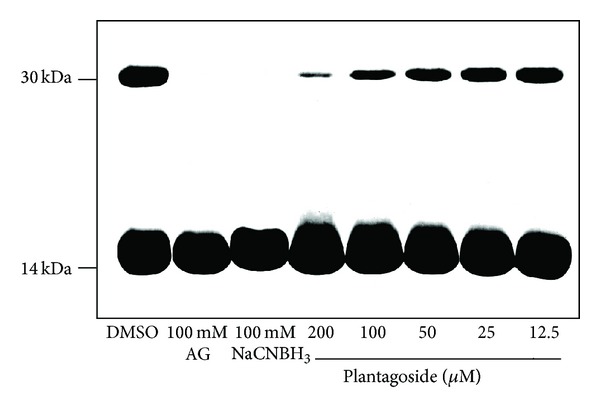
Inhibition of cross-ink formation by plantagoside (**1**). This assay was performed at pH 7.4 in phosphate buffer, which contained 20 mM ribose, 5 mg/mL lysozyme, 100 mM of aminoguanidine or NaCNBH_3_, or the indicated concentration of plantagoside dissolved in dimethylsulfoxide, for 1 week at 37°C. Each sample was then subjected to 17.5% SDS-PAGE and stained with Coomassie Brilliant Blue. DMSO: dimethylsulfoxide containing no inhibitor; AG: aminoguanidine.

For evaluating the inhibitory activity of plantagoside against glycation-dependent cross-link formation, each inhibitor, that is, plantagoside and aminoguanidine, which inhibit cross-link formation by linking with the carbonyl groups of Amadori products [7], or NaCNBH_3_, which inhibits by reducing Amadori products to a secondary amine [19], was incubated at 37°C with lysozyme and ribose in phosphate buffer.  Figure 2 presents the effects of plantagoside against glycation-dependent cross-link formation. A dimer of lysozyme (ca. 29 kDa) was clearly observed after 1 week (lane 1). It was found that 100 mM aminoguanidine (lane 2) and 100 mM NaCNBH_3_ (lane 3) inhibited the dimerization of lysozyme, while 12.5–200 *μ*M plantagoside also caused inhibition in a concentration-dependent manner (lanes 4–8). The results showed that plantagoside can inhibit the formation of protein-protein cross-links via glycation and the formation of AGEs in proteins.

**Figure 3:**
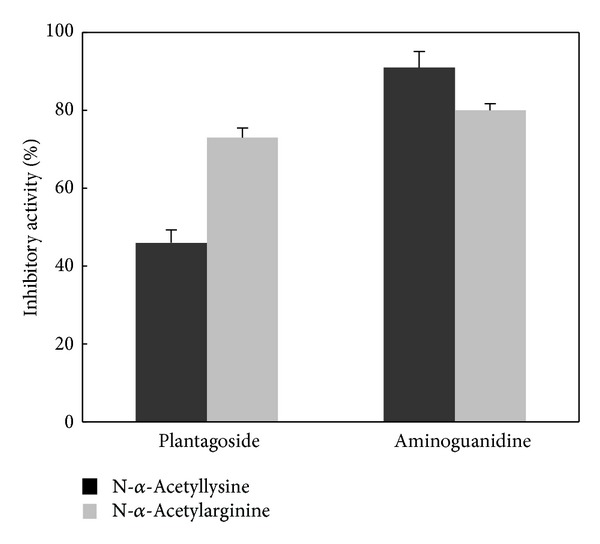
Differences in AGE formation inhibitory activity with different types of amino acids as the substrate. The inhibitory activities were determined at concentrations of 25 *μ*M plantagoside and 10 mM aminoguanidine using N-*α*-acetyllysine or N-*α*-acetylarginine as the substrate for glycation. The inhibitory activity was examined by measuring the increase in the fluorescence intensity because of the formation of AGEs. Each value represents the mean ± standard error of three experiments.

To investigate the change in the inhibitory activity due to the formation of AGEs by plantagoside with different types of amino acids as the substrate, we performed assays using either N-*α*-acetyllysine or N-*α*-acetylarginine. Plantagoside with N-*α*-acetyllysine and N-*α*-acetylarginine as the substrate possessed 46% and 73% inhibitory activities, respectively, (Δ, 27%) at a concentration of 25 *μ*M and strongly inhibited the formation of AGEs with N-*α*-acetylarginine (Figure 3). In contrast, aminoguanidine possessed 91% and 80% of the inhibitory activities (Δ, 11%) at a concentration of 10 *μ*M, while there was no major selectivity of the inhibitory activity. These results indicate that the inhibitory mechanism employed by plantagoside differed from that employed by aminoguanidine.

To develop a more effective inhibitor, we investigated the relationship between the skeletal structures of the aglycones of plantagoside (flavone,** 4**; flavonol,** 6**; flavanone,** 2;** and flavanonol,** 7**; Figure 1) and their inhibitory activity. The results showed that the order of the inhibitory activity of the compounds (highest first) was 5,7,3′,4′,5′-pentahydroxyflavone,** 4** (7.5 *μ*M); myricetin,** 6** (7.9 *μ*M); 5,7,3′,4′,5′-pentahydroxyflavanone,** 2** (18.0 *μ*M); and dihydromyricetin,** 7** (45.0 *μ*M); 5,7,3′,4′,5′-pentahydroxyflavone possessed the highest activity among the aglycones. The results suggested that the putative compound 5,7,4′,5′-tetrahydroxyflavone-3′-*O*-glucoside (**5**) would possess a potent inhibitory activity at a submicromolar concentration, given that the inhibitory activity was 15-times higher after 3′-*O*-glycosylation of 5,7,3′,4′,5′-pentahydroxyflavanone (**2**). Therefore, we intend to synthesize** 5**, which has not been obtained from natural resources.

In this study, we demonstrated the inhibitory activity of plantagoside and its aglycone against the Maillard reaction. In Europe and the USA, the* P. major* seeds that contain these flavanones are known as “Shazenshi,” which is a well-known crude drug. The seed of* P. asiatica* is used as a diuretic in traditional Chinese medicine. There are no reports on the treatment or prevention of diabetic complications using this drug; however, the present study suggests that Shazenshi has potential as a therapeutic agent to combat diabetic complications.

## Abbreviations

BSA: Bovine serum albumin
dd: Double doublet
DMSO: Dimethylsulfoxide
FAB-MS: Fast atom bombardment mass spectrum
glc: Glucose
PBS: Phosphate-buffered saline
SDS-PAGE: Sodium dodecyl sulfate polyacrylamide gel electrophoresis.

## References

[1] Brownlee M, Cerami A, Vlassara H (1988). Advanced glycosylation end products in tissue and the biochemical basis of diabetic complications. *The New England Journal of Medicine*.

[2] Hayase F, Nagaraj RH, Miyata S, Njoroge FG, Monnier VM (1989). Aging of proteins: immunological detection of a glucose-derived pyrrole formed during Maillard reaction in vivo. *Journal of Biological Chemistry*.

[3] Ahmed MU, Thorpe SR, Baynes JW (1986). Identification of N(*ε*)-carboxymethyllysine as a degradation product of fructoselysine in glycated protein. *Journal of Biological Chemistry*.

[4] Dyer DG, Blackledge JA, Thorpe SR, Baynes JW (1991). Formation of pentosidine during nonenzymatic browning of proteins by glucose: Identification of glucose and other carbohydrates as possible precursors of pentosidine in vivo. *Journal of Biological Chemistry*.

[5] Monnier VM, Kohn RR, Cerami A (1984). Accelerated age-related browning of human collagen in diabetes mellitus. *Proceedings of the National Academy of Sciences of the United States of America*.

[6] Obayashi H, Nakano K, Shigeta H (1996). Formation of crossline as a fluorescent advanced glycation end product in vitro and in vivo. *Biochemical and Biophysical Research Communications*.

[7] Brownlee M, Vlassara H, Kooney A (1986). Aminoguanidine prevents diabetes-induced arterial wall protein cross-linking. *Science*.

[8] Huby R, Harding JJ (1988). Non-enzymic glycosylation (glycation) of lens proteins by galactose and protection by aspirin and reduced glutathione. *Experimental Eye Research*.

[9] Booth AA, Khalifah RG, Hudson BG (1996). Thiamine pyrophosphate and pyridoxamine inhibit the formation of antigenic advanced glycation end-products: comparison with aminoguanidine 1. *Biochemical and Biophysical Research Communications*.

[10] Malone JI, Lowitt S, Cook WR (1990). Nonosmotic diabetic cataracts. *Pediatric Research*.

[11] Morimitsu Y, Yoshida K, Esaki S, Hirota A (1995). Protein glycation inhibitors from thyme (*Thymus vulgaris*). *Bioscience, Biotechnology and Biochemistry*.

[12] Matsuura N, Aradate T, Sasaki C (2002). Screening system for the Maillard reaction inhibitor from natural product extracts. *Journal of Health Science*.

[13] Laemmli UK (1970). Cleavage of structural proteins during the assembly of the head of bacteriophage T4. *Nature*.

[14] Endo T, Taguchi H, Yosioka I (1981). The Glycosides of Plantago major var. japonica NAKAI. A New Flavanone Glycoside, Plantagoside. *Chemical & Pharmaceutical Bulletin*.

[15] Forkmann G (1983). 5, 7, 3′, 4′, 5′-pentahydroxyflavanone in the bracts of *Helichrysum bracteatum*. *Zeitschrift für Naturforschung*.

[16] Yamada H, Nagai T, Takemoto N (1989). Plantagoside, a novel *α*-mannosidase inhibitor isolated from the seeds of *Plantago asiatica*, suppresses immune response. *Biochemical and Biophysical Research Communications*.

[17] Ohara M, Kojima H, Matsuura N Maillard reaction products-degrading preparations containing rosmarinic acid, epigallocatechin, etc..

[18] Takahashi H, Kimura K, Yoshihama M, Shinoda K, Negeshi S, Seri K Maillard reaction inhibitors containing aurone derivatives.

[19] Jentoft N, Dearborn DG (1979). Labeling of proteins by reductive methylation using sodium cyanoborohydride. *Journal of Biological Chemistry*.

